# Quantizing Chaplygin Hamiltonizable nonholonomic systems

**DOI:** 10.1038/s41598-022-13335-6

**Published:** 2022-06-08

**Authors:** Oscar E. Fernandez

**Affiliations:** Department of Mathematics, Wellesley College, Wellesley, MA 02482 USA

**Keywords:** Applied mathematics, Quantum physics

## Abstract

In this article we develop a quantization procedure for Chaplygin Hamiltonizable nonholonomic systems—mechanical systems subject to non-integrable velocity constraints whose reduced mechanics is Hamiltonian after a suitable time reparametrization—using Poincaré transformations and geometric quantization. We illustrate the theory developed through examples and discuss potential applications to the study of the quantum mechanics of nanovehicles.

## Introduction

Recent advances in the design and control of nanoscale molecular machines have led to a surge in interest in the quantum mechanics and control of “nanomachines” (see^1^ for a recent review), culminating in the 2016 Nobel Prize in Chemistry for the “design and synthesis of molecular machines”. In some cases experimental evidence has documented nanomachines rolling on graphite and metallic surfaces^2–5^. But while the mechanics of rolling are well-understood at the macroscopic scale—macroscopic rolling systems are *nonholonomic systems* (NHSs, for short): mechanical systems subject to non-integrable velocity constraints—there is no known theory of “quantum nonholonomic mechanics”. A key obstruction to such a theory is the fact that NHSs are not Hamiltonian systems^6^; NHSs equations of motion generally consist of a coupled set of first-order kinematic equations (the nonholonomic constraints) and second-order dynamical equations^7^. However, under certain symmetry conditions—true of so-called *abelian Chaplygin* NHSs—the kinematic equations decouple from the dynamics (now called the *reduced dynamics*), and for *Chaplygin Hamiltonizable* NHSs the reduced dynamics can be transformed into a Hamiltonian system via a smooth reparameterization of time $d\tau =f(q)\,dt$^7–13^. In^14^, it was shown that a Poincaré transformation (from the theory of adaptive geometric integrators^15^[Chap. 9]) accomplishes the same transformation without the need for a time reparametrization. Furthermore, it was shown that the nonholonomic mechanics of the full system (reduced dynamics plus nonholonomic constraints) of Chaplygin Hamiltonizable NHSs are equivalent to the Hamiltonian mechanics of an “associated Hamiltonian system”^16^ provided the initial conditions of that Hamiltonian system satisfy the nonholonomic constraints. This approach was used in^17^ to establish a general quantization scheme for $f(q)=1$ Chaplygin Hamiltonizable NHSs—a class known as *conditionally variational* NHSs^18^—by first quantizing the associated Hamiltonian system using geometric quantization and then enforcing the nonholonomic constraints at the quantum level via a particular choice of the initial wavefunction. In^19^, the same approach was applied to describe the quantum mechanics of a “molecular wheelbarrow”.

In this article we generalize the result of^17^ to establish a quantization scheme for abelian Chaplygin, Chaplygin Hamiltonizable NHSs with non-Euclidean configuration spaces and/or non-unit multipliers. The resulting quantum data features a Hamiltonian operator that, like that found in^17^, contains a term proportional to the Ricci scalar curvature *R* of the kinetic energy metric embedded in the associated Hamiltonian system. But in addition, that quantum operator also depends on the multiplier *f*. The resulting quantum mechanics is thus influenced by a rich interplay of geometry (via *R*), mechanics (via the Hamiltonian of the associated Hamiltonian system), and phase space volume preservation. (Chaplygin Hamiltonizable NHSs preserve phase space volume^7^[ Thm. 8.9.1].) We illustrate our results with examples and discuss the broader potential applications of the work. Because the class of NHSs we study in this article is the most well-studied class of NHSs and include many physical examples of NHSs, we hope that our results will be useful to a variety of other researchers in science and engineering.

## Background

This article deals exclusively with a particular class of mechanical systems known as *abelian Chaplygin* NHSs. To define this class we first define what we mean by a “mechanical system” on a smooth manifold.

### Definition 1

Let *Q* be a smooth, connected, orientable, *n*-dimensional Riemannian manifold with metric *g*. By a **mechanical system on** *Q* we will mean a pair (*Q*, *L*), where $L :TQ \rightarrow {\mathbb {R}}$ is a Lagrangian of mechanical type: $L=T-V$, where $T :TQ \rightarrow {\mathbb {R}}$ is the kinetic energy given by $T(q,{\dot{q}})=\frac{1}{2}g_{ij}(q){\dot{q}}^{i}{\dot{q}}^{j}$, $i,j=1,\ldots ,n$ (here $g_{ij}$ are the components of *g*) and $V: Q \rightarrow {\mathbb {R}}$ is the potential energy (we identify *V* with its lift to *TQ*), and is assumed to be a smooth function.

(We hereafter adhere to the Einstein summation convention for repeated indices.)

Define now a *constraint distribution* ${{\mathscr {D}}} \subset TQ$ by the one-forms $\{\omega ^{a}\}_{a=1}^{k}$, $k<n$, as ${{\mathscr {D}}} = \{v \in TQ \,|\, \omega ^{a}(v)=0, \,\, a=1,\ldots ,k\}$. If we assume that the constraints are linear and homogeneous—so that locally $\omega ^{a}(v)=c^{a}_{j}(q){\dot{q}}^{j}$—and that ${{\mathscr {D}}}$ has constant rank, then the triple $(Q,L,{{\mathscr {D}}})$ becomes a **nonholonomic mechanical system**^7^.

Now, suppose that a *k*-dimensional Lie group *G* acts freely and properly on *Q*, so that ${\overline{Q}}:=Q/G$ is a manifold. Let $\mathfrak {g}$ be the Lie algebra of *G*, and $\xi _{Q}$ the infinitesimal generator on *Q* corresponding to $\xi \in \mathfrak {g}$.

We assume that both *L* and ${{\mathscr {D}}}$ are *G*-variant with respect to the lifted action, and that at each $q \in Q$, $T_{q}Q=\mathfrak {g}_{Q} \oplus {{\mathscr {D}}}_{q}$, where $\left. \mathfrak {g}_{Q}\right| _{q}=\{\xi _{Q}(q)\,|\,\xi \in \mathfrak {g}\}$ is the tangent to the orbit through $q \in Q$^7^[Section 2.8]. Then we will call $(Q,L,{{\mathscr {D}}},G)$ a **Chaplygin nonholonomic mechanical system**^7^.

### Definition 2

An **abelian Chaplygin nonholonomic system** is a Chaplygin nonholonomic mechanical system $(Q,L,{{\mathscr {D}}},G)$ such that:

1. $G={\mathbb {R}}^l \times {\mathbb {S}}^{k-l}$ *(*with $0 \le l \le k$*)*, and thus $q \in Q$ can be decomposed as $q=(r^{\alpha },s^a)$, where $\alpha =1,\ldots ,n-k$, $a=1,\ldots ,k$;
2. The nonholonomic constraints are of the form ${\dot{s}}^a=-A^{a}_{\alpha }(r){\dot{r}}^{\alpha }$;

We note that the $r^{\alpha }$ are the ${\overline{Q}}$-coordinates, the $s^{a}$ are invariant coordinates (the *G*-coordinates), and, as a consequence of the assumptions above, both *L* and the nonholonomic constraints are independent of these *s*-variables. (We hereafter restrict all Greek and Latin indices to the same range as $\alpha$ and *a* above, respectively.)

The equations of motion of an abelian Chaplygin NHS consist of a system of second-order ordinary differential equations on *Q* (the *reduced equations*, (1a) below; see^7^[Sec. 5.4] for details of the reduction process) together with the (first-order) nonholonomic constraints, (1b) below:

1a $$\begin{aligned}&\displaystyle E_{\alpha }(L_c)=S_{\alpha \beta }{\dot{r}}^{\beta }, \quad \text {where} \quad S_{\alpha \beta }=-\left( \frac{\partial L}{\partial {\dot{s}}^{a}}\right) ^*\left( \frac{\partial A^{a}_{\alpha }}{\partial r^{\beta }}-\frac{\partial A^{a}_{\beta }}{\partial r^{\alpha }}\right) , \end{aligned}$$

1b $$\begin{aligned}&\displaystyle {\dot{s}}^{a}=-A^{a}_{\alpha }(r){\dot{r}}^{\alpha }, \end{aligned}$$

where $L_c(r,{\dot{r}})=L(r,{\dot{r}},{\dot{s}}=-A\cdot {\dot{r}}){:T{\overline{Q}} \rightarrow {\mathbb {R}}}$ is the *constrained Lagrangian*, $E_{\alpha }=\frac{d}{dt}\frac{\partial }{\partial {\dot{r}}^{\alpha }}-\frac{\partial }{\partial r^{\alpha }}$, and the asterisk ($^*$) in (1a) indicates that we substitute (1b) into (1a) *after* differentiation.) We will refer to the full set of equations (1) as the *Lagrange-d’Alembert equations*^7^[Sec. 5.2].

Now, although the Lagrange-d’Alembert equations (1) are not Hamiltonian, as discussed in the Introduction the reduced equations (1a) can sometimes be transformed into Hamiltonian form. Specifically, suppose there exists a smooth non-vanishing “multiplier” $f(r):{\overline{Q}} \rightarrow {\mathbb {R}}$ such that after the reparameterization $d\tau =f(r)\,dt$ of (1a) the gyroscopic terms $S_{\alpha \beta }{\dot{r}}^{\beta }$ vanish. We then call the system **Chaplygin Hamiltonizable**, since in $\tau$-time the reduced equations (1a) are Hamiltonian. In^14^, we showed that for such systems, if we: (a) select initial conditions $r(0)=r_0$ and ${\dot{r}}(0)={\dot{r}}_0$, calculate the corresponding constrained energy value ${{\mathscr {E}}}^0_c(r_0,{\dot{r}}_0):={\dot{r}}^{\alpha }(\partial L_c/\partial {\dot{r}}^{\alpha })-L_c$ (which is conserved for NHSs), construct the Lagrangian $L_p: TQ/G \rightarrow {\mathbb {R}}$,

2 $$\begin{aligned} L_{p}(r,{\dot{r}},{\dot{s}}) = f(r)\left[ L(r,{\dot{r}},{\dot{s}})-\frac{\partial L}{\partial {\dot{s}}^a}{(r,{\dot{r}},{\dot{s}})}\phi ^{a}{(r,{\dot{r}},{\dot{s}})}\right] +f(r){{\mathscr {E}}}^0_c, \quad \text {where} \quad \phi ^{a}(r,{\dot{r}},{\dot{s}})={\dot{s}}^a+A^{a}_{\alpha }(r){\dot{r}}^{\alpha }, \end{aligned}$$

and (b) select ${\dot{s}}(0)={\dot{s}}_0$ such that $\phi ^{a}(r_0,{\dot{r}}_0,{\dot{s}}_0)=0$, then

the Euler-Lagrange equations of $L_p$ become the Lagrange-d’Alembert equations (1) when restricted to the energy level set ${{\mathscr {E}}}^0_c$ from (a). (An explicit example of this is contained in the discussion surrounding^19^[Eqn. (9)].) In coordinates,

$$\begin{aligned} L_p(r,{\dot{r}},{\dot{s}})=\left[ \frac{1}{2}f(r)\left\{ g_{ij}(r){\dot{q}}^{i}{\dot{q}}^{j}-2g_{aj}(r){\dot{s}}^{a}{\dot{q}}^{j}-2g_{aj}(r)A^{a}_{\alpha }(r){\dot{r}}^{\alpha }{\dot{q}}^{j}\right\} \right] -f(r)\left( V(r)-{{\mathscr {E}}}^0_c\right) , \end{aligned}$$

and thus (2) is of mechanical type with kinetic energy given by the bracketed term and potential energy $f(r)\left( V-{{\mathscr {E}}}^0_c\right)$. These results motivate the following definition. (Henceforth we assume, as per our definition above of “Chaplygin Hamiltonizable”, that all associated multipliers are smooth and non-vanishing.)

### Definition 3

Let $(Q,L,{{\mathscr {D}}}, G)_{f}$ be an abelian Chaplygin nonholonomic system that is also Chaplygin Hamiltonizable with multiplier *f*, and suppose that $L_p$ and the constrained Lagrangian $L_c$ are both regular. We will then refer to $(Q,H_p,G)$, where $H_p$ is the Hamiltonian corresponding to (2), as the **associated Hamiltonian system** to $(Q,L,{{\mathscr {D}}},G)_f$.

Our strategy to quantize $(Q,L,{{\mathscr {D}}},G)_f$ is now as follows: (i) describe the Hamiltonian system $(Q,H_p,G)$, (ii) quantize it via geometric quantization (needed since (*Q*, *g*) is generally not a Euclidean space with a Euclidean metric), and (iii) impose the Hamiltonian analogues of the aforementioned initial conditions and impose the energy level set restriction at the quantum level. Let us now briefly review the relevant results from geometric quantization before executing this strategy in the next section.

The geometric quantization of a mechanical system (*Q*, *L*) proceeds as follows (see^20–23^ for thorough expositions of geometric quantization, and^17^[Appendix A] for a summary of the main results relevant to our purposes). First, one verifies the following prequantization requirements:

1. *Q* is a connected, orientable, and smooth Riemannian manifold with respect to *g* (the kinetic energy metric of *L*);
2. *Q* is complete with respect to the metric induced by *g*;
3. The Hamiltonian vector field $X_{H}$, where *H* is the Hamiltonian corresponding to *L*, is a complete vector field.

If these are met, then (*Q*, *L*) is quantizable in the Schrödinger representation (the representation in which the prequantization’s line bundle is trivial—so that, by fixing a global trivializing section, any section of this bundle can be identified with functions on $T^*Q$ (the wavefunctions)—and the vertical polarization is selected to yield solely *q*-dependent wavefunctions) with the following quantum data^20–22^:

- The Hilbert space ${{\mathscr {H}}} \cong L^2(Q,\sqrt{\det g})$ consists of wavefunctions $\psi \in C^{\infty }(Q,{\mathbb {C}})$;
- The (self-adjoint) quantum operators for the position and momenta are the standard operators (${\hat{q}}^i=q^i$ and ${\hat{p}}_i = -i\hslash \partial /\partial q^i$), and the Hamiltonian operator—calculated in^22^[Sec. 9.7] and^23^[Chapter 9]—is given by
  3 $$\begin{aligned} {\widehat{H}}=-\frac{\hslash ^2}{2}\left( \Delta - \frac{R}{6}\right) +V, \end{aligned}$$
  where $\Delta$ is the Laplace-Beltrami operator, *R* is the Ricci scalar curvature of the metric *g*, and *V* is the potential of the Lagrangian *L* associated with the Hamiltonian *H*.

## Results

We now execute the strategy outlined after Definition 3. We start by addressing part (i) of that strategy.

### Theorem 1

*Let* $(Q,L,{{\mathscr {D}}}, G)_f$ *be an abelian Chaplygin nonholonomic system that is also Chaplygin Hamiltonizable with smooth multiplier* *f*(*r*); $r(0)=r_0$, ${\dot{r}}(0)={\dot{r}}_0$ *be an initial condition for the system* (1a) *with associated constrained energy* ${{\mathscr {E}}}^0_c$; *and* $(Q,H_p,G)$ *be the associated Hamiltonian system. Then the* Lagrange-d’Alembert equations (1) *are equivalent to the Hamiltonian mechanics of* *the Hamiltonian* $H_p: T^*Q/G \rightarrow {\mathbb {R}}$ *given by*

4 $$\begin{aligned} H_p(r,p_r,p_s)=f(r)\left( H_c(r,p_r)-{{\mathscr {E}}}^0_c\right) +f(r)\left( L_c(r,{\dot{r}})-L(r,{\dot{r}},{\dot{s}})\right) \quad \left. \right| _{(q,{\dot{q}}) \mapsto (q,p)}, \end{aligned}$$

*provided* $p_a(0)=0$, *where* $p_a=\partial L_p/\partial {\dot{s}}^a$, *and that we restrict the mechanics to the fixed energy value* ${{\mathscr {E}}}^0_c$. *In* (4) $H_c: T^*{{\overline{Q}}} \rightarrow {\mathbb {R}}$ *is the* *constrained Hamiltonian* *associated with the constrained Lagrangian* $L_c$.

### Proof

For part 1, from (2) we have:

5 $$\begin{aligned} p_{\alpha }= & {} \frac{\partial L_p}{\partial {\dot{r}}^{\alpha }} = f(r)\left( \frac{\partial L}{\partial {\dot{r}}^{\alpha }}-g_{\alpha a}\phi ^{a}-\frac{\partial L}{\partial {\dot{s}}^{a}}A^{a}_{\alpha }\right) = f(r)\frac{\partial L_c}{\partial {\dot{r}}^{\alpha }}-f(r)g_{\alpha a}\phi ^{a} \end{aligned}$$

6 $$\begin{aligned} p_a= & {} \frac{\partial L_p}{\partial {\dot{s}}^{a}} = -f(r)g_{ab}\phi ^{b}, \end{aligned}$$

where we used^7^[Eqn. (5.8.25)] to get the last equality in (5). Then, from the Legendre transform $H_p={\dot{q}}^i\frac{\partial L_p}{\partial {\dot{q}}^i}-L_p={\dot{r}}^{\alpha }p_{\alpha }+{\dot{s}}^{a}p_a-L_p$, substituting in (5), (6), and (2) yields:

7 $$\begin{aligned} H_p = f(r){\dot{r}}^{\alpha }\frac{\partial L_c}{\partial {\dot{r}}^{\alpha }}-f(r)g_{\alpha a}{\dot{r}}^{\alpha }\phi ^{a}-f(r)g_{ab}{\dot{s}}^{a}\phi ^{b}-f(r)\left[ \left( L+{{\mathscr {E}}}^0_c\right) -\frac{\partial L}{\partial {\dot{s}}^{a}}\phi ^{a}\right] . \end{aligned}$$

Using now the fact that ${\dot{r}}^{\alpha }\frac{\partial L_c}{\partial {\dot{r}}^{\alpha }}-L_c=H_c \implies {\dot{r}}^{\alpha }\frac{\partial L_c}{\partial {\dot{r}}^{\alpha }}=H_c+L_c$, (7) becomes

8 $$\begin{aligned} H_p = f(r)\left( H_c-{{\mathscr {E}}}^0_c\right) +f(r)\left( L_c-L\right) +f(r)\left[ \frac{\partial L}{\partial {\dot{s}}^{a}}-g_{\alpha a}{\dot{r}}^{\alpha }-g_{ba}{\dot{s}}^{b}\right] \phi ^{a}. \end{aligned}$$

But $L=\frac{1}{2}g_{ij}{\dot{q}}^i{\dot{q}}^j-V$ (from Definition 1) implies $\frac{\partial L}{\partial {\dot{s}}^{a}}=g_{a\alpha }{\dot{r}}^{\alpha }+g_{ab}{\dot{s}}^{b}=g_{\alpha a}{\dot{r}}^{\alpha }+g_{ba}{\dot{s}}^{b}$, since *g* is symmetric (*g* is a Riemannian metric). Thus the bracketed term in (8) vanishes, yielding (4). (We left the last term in (4) untransformed since it vanishes when the nonholonomic constraints are imposed.)

For part 2, since *G* is abelian and acts (freely and properly) on *Q* (by translation on the *s* variables), it induces an action of *G* on $T^*Q$. The associated momentum map^7^ $J: T^*Q \rightarrow {\mathfrak {g}}^*$ has components $J_a(q,p)=p_a$. Clearly, $H_p$ is also *G*-invariant (the *s* variables are cyclic), and thus from Noether’s Theorem^24^ it follows that that the $p_a$ are conserved. From (6) we thus have:

9 $$\begin{aligned} p_a = -f(r)g_{ab}(r)\phi ^{b}=\mu _a \implies {\dot{s}}^{a}+A^{a}_{\alpha }(r){\dot{r}}^{\alpha }=-\frac{g^{ab}(r)}{f(r)}\mu _b, \end{aligned}$$

where $g^{ab}(r)$ is the inverse matrix of $g_{ab}(r)$ (recall we assumed in Definition 1 that *L* is regular, so that $g_{ab}(r)$ is invertible; recall also that *f* is non-vanishing by assumption). Now, since we have assumed the action of *G* to be free it follows that every $\mu \in {\mathfrak {g}}^*$ is a regular value of *J*^20^[Prop. 2.2]. Thus, for any $\mu \in {\mathfrak {g}}^*$ the *reduced space* $J^{-1}(\mu )/G=T^*{\overline{Q}}$ (recall that ${\overline{Q}}=Q/G$)^24^. For the zero level set of *J*, the reduced space $T^*{\overline{Q}}$ always carries the canonical symplectic form^24^. Moreover, the *reduced Hamiltonian* $h_p: T^*{\overline{Q}} \rightarrow {\mathbb {R}}$ is defined by

$$\begin{aligned} h_p \circ \pi _{\mu }=H_p \circ i_{\mu }, \end{aligned}$$

where $\pi _{\mu }:J^{-1}(\mu ) \rightarrow T^*{\overline{Q}}$ is the canonical projection and is the inclusion^21^; simply put, $h_p$ is just (4) with $p_a=\mu _a$, $a=1,\ldots ,k$, which with the help of (9) yields

10 $$\begin{aligned} h_p(r,p_r,\mu )=f(r)\left( H_c(r,p_r)-{{\mathscr {E}}}^0_c\right) +f(r)\left( L_c(r,{\dot{r}})-L(r,{\dot{r}},{\dot{s}})\right) \quad \left. \right| _{(r,{\dot{r}},{\dot{s}}) \mapsto (r,p_r,p_s) \mapsto (r,p_r,\mu )}. \end{aligned}$$

Thus, the Hamiltonian mechanics of $H_p$ is just the Hamiltonian mechanics of $h_p$ together with the conservation laws $p_a(t)=\mu _a$. Let us now see how this system reproduces (1).

To the initial conditions $r(0)=r_0$, ${\dot{r}}(0)={\dot{r}}_0$ already been imposed in the theorem statement, let us now add the choice of initial condition ${\dot{s}}(0)=-A(r_0) \cdot {\dot{r}}_0$. This implies $\phi ^{a}(0)=0$. (One can calculate the corresponding initial momenta conditions from (5)–(6).) The first equation in (9) then yields $p_a(0)=0=\mu _a$, and using $\mu _a=0$ in the second equation in (9) implies $\phi ^{a}=0$, so that the nonholonomic constraints (1b) are satisfied throughout the mechanics of the Hamiltonian system $(Q,H_p,G)$. Next, since the choice of $\mu _a=0$ enforces the nonholonomic constraints, when $\mu _a=0$ the second term in (10) vanishes (since we recall that $L_c(r,{\dot{r}})=L(r,{\dot{r}},{\dot{s}}=-A \cdot {\dot{r}})$). Thus, $\left. h_p\right| _{\mu _a=0}=:H_{c,p}$, where

11 $$\begin{aligned} H_{c,p}(r,p_r)=f(r)\left( H_c(r,p_r)-{{\mathscr {E}}}^0_c\right) . \end{aligned}$$

In^14^[Thm. 1, part 1] we showed that the nonholonomic dynamics (1a) is equivalent to the Lagrangian mechanics of the Lagrangian $L_{c,p}(r,{\dot{r}})= f(r)(L_c(r,{\dot{r}})+{{\mathscr {E}}}^0_c)$. And since the Legendre transform of $L_p$ is $H_p$, we conclude that the Hamiltonian mechanics of $H_p$ reproduces the nonholonomic dynamics (1a). $\square$

We now proceed to part (ii) of the strategy outlined after Definition 3. The theorem below furnishes sufficient conditions for the verification of the prequantization requirements (i)–(iii) from Section 1.

### Theorem 2

*Let* (*Q*, *L*) *be a mechanical system, with dim*$(Q)=n$, *and denote by* *g* *the Riemannian metric of the kinetic energy term of* *L*. *Suppose that*:

1. $T_qQ \cong {\mathbb {R}}^n$ for each $q \in Q$ $\mathrm {(}$*i.e., each tangent space to* *Q* *is isomorphic to n-dimensional Euclidean space*$\mathrm {)}$;
2. *Q* *is complete with respect to the Euclidean metric*;
3. *There exist positive constants* *a*, *b* *such that* $a \le \lambda _i(q) \le b$ *for all* $i=1,\ldots ,n$ *and for all* $q \in Q$, *where* $\lambda$ *are the eigenvalues of* *g*;
4. *The potential function* $V \ge 0$ *for all* $q \in Q$.

*Then*: $\mathrm {(i)}$ $(Q,d_g \mathrm {)}$ *(*where $d_{g}$ *is the metric on* *Q* *induced by* *g)* *is complete, and* $\mathrm {(ii)}$ *the Hamiltonian vector field* $X_H$, *where* *H* *is the Hamiltonian associated with* *L*, *is a complete vector field.*

### Proof

For part (i), that (*Q*, *L*) is a mechanical system implies, via Definition 1, that *Q* is connected and *g* is a Riemmanian metric. It follows from^25^[Prop. 7.2.5] that *g* induces a metric space structure on *Q*, with the induced distance^25^[Section 7.2] being the infimum of

12 $$\begin{aligned} \ell _g(\gamma ):=\int _{a}^{b}\sqrt{g(\gamma '(t),\gamma '(t))}\,dt, \end{aligned}$$

the length of a piecewise differentiable path connecting *p* and *q*, where $p,q \in Q$ and $\gamma :[a,b] \rightarrow Q$. Thus, $(Q,d_g)$ is a metric space. We now prove that this space is complete. First, fix $q \in Q$. From assumption #1, $v \in T_qQ \cong {\mathbb {R}}^n$. Then *g*(*v*, *v*) is a quadratic form we denote by *F*: $F(v)=g(v,v)=v^TMv$, where *M* is the Hessian of *L* and $v=(v_1,\ldots ,v_n)$, with $v_i$ the *i*-th component of the vector *v*^26^[Section V.7]. We now follow the proof of^17^[Theorem 3]. Namely, since *g* is a Riemannian metric, *M* is a positive-definite and symmetric matrix. It follows from the Principal Axes Theorem^27^[Chapter X, Theorem 19] that *M* is orthogonally diagonalizable, that is, there is an orthogonal matrix *O* such that $O^TMO=D$, where *D* is the diagonal matrix of eigenvalues of *M*. As a consequence, if $\lambda _1(q),\ldots ,\lambda _n(q)$ are the eigenvalues of *M* then in the new variable $y=O^Tv$ (or $v=Oy$) we have $F(v)=F(Oy)=g(Oy,Oy)=y^TO^TMOy=y^TDy=\lambda _i(q)y^2_i.$ Assumption #3 then implies that

13 $$\begin{aligned} ay^Ty \le F(v) \le by^Ty \quad \iff \quad av^Tv \le F(v) \le bv^Tv \quad \iff \quad a||v||^2_e \le F(v) \le b||v||^2_e, \end{aligned}$$

where $||v||_e$ denotes the norm of *v* with respect to the Euclidean metric $g_e$ on *Q*. Since $q \in Q$ was arbitrary this inequality is true for all $q \in Q$. Following again the proof of^17^[Theorem 3], if we denote by $d_{g_e}$ the distance induced by the Euclidean metric $g_e$ on $T_qQ \cong {\mathbb {R}}^n$ (the usual Pythagorean distance), then using (13) in (12) implies that $ad_{g_e}(p,q) \le d_{g}(p,q) \le bd_{g_e}(p,q)$ for any $p,q \in Q$. Thus, every Cauchy sequence in the metric space $(Q,d_{g})$ is also a Cauchy sequence in the metric space $(Q,d_{g_e})$. And since by assumption #2 $(Q,d_{g_e})$ is complete, it follows that $(Q,d_{g})$ is also complete.

For part (ii), having just shown that $(Q,d_{g})$ is a complete Riemannian manifold, and recalling assumption #4 (that $V \ge 0$), both assumptions from part (ii) of the theorem in^28^ are satisfied, and thus it follows from that theorem that $X_{H}$ is a complete vector field. $\square$

We continue part (ii) of our quantization strategy with the theorem below, which quantizes the associated Hamiltonian system.

### Theorem 3

*Let* $(Q,L,{{\mathscr {D}}},G)_f$ *be an abelian Chaplygin nonholonomic system that is also Chaplygin Hamiltonizable* *with multiplier* *f*, *with* $(Q,H_p,G)$ *its associated Hamiltonian system, and let* $r(0)=r_0$, ${\dot{r}}(0)={\dot{r}}_0$ *be an initial condition with associated constrained energy* ${{\mathscr {E}}}^0_c$. *Denote by* $g_p$ *the kinetic energy metric of the Lagrangian* $L_p$ *from* (2) *and suppose also that* $(Q,L_p)$ *satisfies the hypotheses of Theorem*2. *Then* $(Q,H_p,G)$ *is quantizable in the* *Schr*$\ddot{o}$*dinger representation with the following quantum data*:

1. The *(self-adjoint) quantum operators for the position and momenta are the standard operators* *(*${\hat{q}}^i=q^i$ *and* ${\hat{p}}_i = -i\hslash \partial /\partial q^i$*)*, *and the Hamiltonian operator is given by*
  14 $$\begin{aligned} {\widehat{H}}_p=-\frac{\hslash ^2}{2}\Delta +\left[ \frac{R\hslash ^2}{12}+f(r)(V-{{\mathscr {E}}}^0_c)\right] , \end{aligned}$$
  *where* $\Delta$ *is the Laplace–Beltrami operator*, *R* *is the Ricci scalar curvature of the metric* $g_p$, and *V* *is the potential function of the Lagrangian* *L* *in* $L_p$ *from* (2);
2. *The Hilbert space* ${{\mathscr {H}}} \cong L^2(Q,\sqrt{\det g_p})$ *consists of wavefunctions* $\psi \in C^{\infty }(Q,{\mathbb {C}})$ *of the form* $\psi (q)=\psi _r(r)e^{\frac{i}{\hslash }\mu _as^a}$.

### Proof

The hypotheses, along with Definitions 1–3, imply that all three prequantization requirements of “Introduction” are satisfied by the mechanical system $(Q,L_p)$. Therefore, from (a) and (b) leading up to (3) we know that ${{\mathscr {H}}} \cong L^2(Q,\sqrt{\det g_p})$, the standard quantum operators for *q* and *p* hold, and the Hamiltonian operator is given by (3) applied to $H_p$. To that point, from (2) we see that the potential function of $L_p$ is $f(r)\left( V-{{\mathscr {E}}}^0_c\right)$, where *V* is the potential function of *L*. Thus, (3) applied to $H_p$ yields (14). Finally, since $H_p$ is independent of *s* (recall (4)) the operators ${\hat{p}}_a$ commute with ${\widehat{H}}_p$, and therefore they share a basis of simultaneous eigenfunctions. For $\psi \in {{\mathscr {H}}}$, the eigenfunction equations ${\hat{p}}_a(\psi )=\mu _a\psi$ become $-i\hslash \frac{\partial \psi }{\partial s^a}=\mu _a\psi$, whose solutions are $\psi (q)=\psi _r(r)e^{\frac{i}{\hslash }\mu _as^a}$. $\square$

We are now ready to execute part (iii) of our quantization strategy. Recall from the lead-up to (11) that setting $\mu _a=0$ enforced the nonholonomic constraints throughout the mechanics. In the quantum setting, since the $\mu _a$ are the eigenvalues of ${\hat{p}}_a$, to enforce the constraints at the quantum level we follow^19^ and choose an initial time-dependent wavefunction $\Psi _0(q):=\Psi (q,t=0)$ such that

15 $$\begin{aligned} 0=\langle {\hat{p}}_a\rangle _{\Psi _0} =\langle \Psi _0(q), {\hat{p}}_a(\Psi _0(q))\rangle =-i\hslash \int \Psi _0(q)\left[ \frac{\partial \overline{\Psi _0}(q)}{\partial s^a}\right] \sqrt{\det g_p}\,d^nq. \end{aligned}$$

(Note that any $\Psi _0(q)$ independent of *s* will satisfy (15).) Thus, the nonholonomic constraints are only imposed *on average* at the quantum level. Finally, let us discuss how to restrict the quantum mechanics to the energy level set ${{\mathscr {E}}}^0_c$. From the initial Schrödinger equation ${\widehat{H}}_p(\psi )=E(\mu )\psi$ with energy $E(\mu )$, the choice of $\Psi _0(q)$ in (15) yields a new energy value ${\widetilde{E}}:=E(0)$ (i.e., ${\widetilde{E}}$ is $E(\mu )$ with $\mu _a=0$). To restrict the quantum mechanics to the energy level set ${{\mathscr {E}}}^0_c$ we simply now demand that ${\widetilde{E}}={{\mathscr {E}}}^0_c$. Solving ${\widehat{H}}_p(\psi )={{\mathscr {E}}}^0_c\psi$, with the initial wavefunction choice (15), will therefore both restrict the quantum mechanics to the energy level set ${{\mathscr {E}}}^0_c$ and satisfy the quantum version of the nonholonomic constraints (on average).

We close this section with a note about the configuration spaces that Theorem 3 applies to. We first note that Theorem 3 assumes that we are dealing with an abelian Chaplygin NHS. From Definition 2 this means that the system’s configuration space is $Q={\overline{Q}} \times {\mathbb {R}}^l \times {\mathbb {S}}^{k-l}$. Next, since Theorem 3 assumes that the hypotheses of Theorem 2 are satisfied, hypotheses 1 and 2 of that theorem impose additional restrictions on *Q*, and in particular on ${\overline{Q}}$. Two large classes for which these additional restrictions are satisfied are: ${\overline{Q}}_1={\mathbb {R}}^{n-k}$ and ${\overline{Q}}_2={\mathbb {R}}^{p}\times {\mathbb {S}}^{(n-k)-p}$ (here $0 \le p \, {<}\, n-k$). The resulting tangent spaces $T_qQ_1$ and $T_qQ_2$ are clearly Euclidean, and since both $Q_1$ and $Q_2$ are products of complete spaces, they are complete.

## Examples

We begin first with all the examples—and classes of examples—of NHSs quantized in^17^. These all satisfy the hypotheses of Theorem 3 and feature $f(r)=1$. But because the results of^17^ required $Q={\mathbb {R}}^n$, our results herein extend those quantizations to the more configuration spaces satisfying Theorem 3. In particular, this includes the more general reduced configuration spaces ${\overline{Q}}={\mathbb {R}}^{p}\times {\mathbb {S}}^{n-p}$. Such (reduced) configuration spaces often arise from the presence of angular variables in the physical system modeled by the NHS. Thus, the quantizations achieved in^17^ can now be extended to these new contexts.

Next, we note that there is a large literature on Chaplygin Hamiltonizable NHSs featuring $f(r) \ne 1$. For example, in^29^ the authors detail, in a handy table, a variety of cases in which a rigid body rolling on a plane or sphere is Chaplygin Hamiltonziable, some including potentials and some not. For these and related Chaplygin Hamiltonizable NHSs—see^14^ and references therein for additional references for Chaplygin Hamiltonizable NHSs—there are likely to be classes of the parameters involved which satisfy the hypotheses of Theorem 3. (This is what occurred, for example, in the NHS studied in^19^.) As an explicit example of this, consider the class of abelian Chaplygin NHSs studied in^16^ whose Lagrangian and constraints are given by

16 $$\begin{aligned} L=\frac{1}{2}\left( I_1({\dot{r}}^1)^2+I_2({\dot{r}}^2)^2+I_{a}({\dot{s}}^{a})^2\right) , \quad {\dot{s}}^{a}=-A^{a}_{2}(r^1){\dot{r}}^2, \end{aligned}$$

where $I_1,I_2,I_a$ are constants and $(r^1,r^2,s^a) \in Q$. As shown in^16^, these systems are Chaplygin Hamiltonizable with multiplier

17 $$\begin{aligned} f(r^1)=\frac{1}{\sqrt{I_2+I_{a}(A^{a}_2)^2}}. \end{aligned}$$

This class includes many physical and well-studied examples of NHSs—including the “nonholonomic free particle”^7^[Sec. 5.6.2], the vertically rolling disk^7^[Sec. 1.4], and the mobile robot^30^. The Lagrangian (2) was calculated in^14^ as:

18 $$\begin{aligned} L_{p}=\frac{1}{\sqrt{I_2+I_{a}(A^{a}_2)^2}}\left\{ \frac{1}{2}\left[ I_1({\dot{r}}^1)^2+I_2({\dot{r}}^2)^2-I_a({\dot{s}}^a)^2\right] -I_aA^{a}_2{\dot{s}}^a{\dot{r}}^2+{{\mathscr {E}}}^0_c\right\} . \end{aligned}$$

We now illustrate how one can determine the set of parameters for which the hypotheses of Theorem 3 are satisfied for the special case of $a=1$. (This leads to $\text {dim}\,Q=3$.) For ease of exposition we use set $(r^1,r^2,s^1)=(x,y,z)$. First, we choose *Q* to satisfy the hypotheses of Theorem 3. For example, $Q={\mathbb {R}}^3$, $Q={\mathbb {R}}^2 \times {\mathbb {S}}^{1}$, $Q={\mathbb {R}} \times {\mathbb {S}}^{1} \times {\mathbb {S}}^{1}$ and similar would work. Then parts 1 and 2 of Theorem 2 are satisfied. Since $V=0$ in (18) then part 4 is satisfied as well. Four checks remain: (a) that *f* is non-vanishing and smooth; (b) that the kinetic energy metric of (18),

19 $$\begin{aligned} g_p = \begin{pmatrix} f(x)I_1 &{} 0 &{} 0 \\ 0 &{} f(x)I_2 &{} -f(x)I_3A^{z}_{y}(x) \\ 0 &{} -f(x)I_3A^{z}_{y}(x) &{} -f(x)I_3 \end{pmatrix}, \end{aligned}$$

is positive-definite (this is required to meet Definition 1), (c) that part 3 of Theorem 2 is also satisfied, and (d) that (18) and the constrained Lagrangian associated with *L* from (16) are regular. We now investigate these.

- The determinants $d_i$ of the upper-left $l \times l$ submatrices (where $l=1,2,3$) of (19) are
  $$\begin{aligned} d_1=f(x)I_1, \quad d_2=\left[ f(x)\right] ^2I_1I_2, \quad d_3=-f(x)I_1I_3. \end{aligned}$$
  If $I_1>0$, $I_2>0$, and $I_3<0$ then all $d_i$ are positive. It follows in this case from Sylvester’s criterion^31^[Theorem 7.2.5] that (19) is positive definite.
- For (17) to be non-vanishing (and real-valued) we need $I_2+I_3\left( A^{z}_y(x)\right) ^2>0$. Recalling that $I_3<0$ (from (a)), if we assume that $I_2 = -kI_3$, with $k>1$, then this requirement is satisfied when
  20 $$\begin{aligned} k>\left( A^{z}_y(x)\right) ^2. \end{aligned}$$
  This necessarily implies that $A^{z}_y(x)$ is a bounded function. Additionally, for *f* to be smooth we need this function to be smooth.
- The eigenvalues of (19) are
  21 $$\begin{aligned} \lambda _1=f(x)I_1, \quad \lambda _{\pm }=\frac{I_2-I_3 \pm \sqrt{\delta }}{2g(x)}, \quad \text {where} \quad g(x)=\frac{1}{f(x)}, \quad \delta =(I_3-I_2)^2+4\left[ g(x)\right] ^2I_3. \end{aligned}$$
  Then $\lambda _1$ is uniformly bounded provided there exist positive constants $a_1>b_1$ such that
  22 $$\begin{aligned} \frac{1}{a_1} \le f(x)I_1 \le \frac{1}{b_1}. \end{aligned}$$
  To ensure that $\delta$ is real we need $\delta \ge 0$. This requires that $(I_3-I_2)^2+4\left[ g(x)\right] ^2I_3 \ge 0$. Since $I_3<0$ (from (a)) and $I_2=-kI_3$ (from (b)), this leads to the requirement
  23 $$\begin{aligned} -I_3 \ge \left( \frac{b_1I_1}{k+1}\right) ^2. \end{aligned}$$
  Then, since $I_2>I_3$ (which follows from $I_2=-kI_3$, $I_3<0$, and $k>1$), and since $4[g(x)]^2I_3<0$ (since $I_3<0$ and $g(x)>0$) implies that $I_2-I_3-\sqrt{\delta }>0$, it follows that $\lambda _{-} >0$. And since $\lambda _{+} \ge \lambda _{-}$, it follows that $\lambda _{+} >0$. Therefore, there exist uniform lower bounds for $\lambda _{\pm }$. It remains to find a uniform upper bound for $\lambda _{+}$. (Then $\lambda _{-}$ will be uniformly bounded by the same bound, since $\lambda _{-} \le \lambda _{+}$.) To do so, we simply use the fact that (22) and $I_2=-kI_3$ imply that $\delta \le (k+1)^2I^2_3+4a^2_1I_1^2I_3$. This, along with (22) (converted into bounds for *g*(*x*)) imply that $\lambda _{+}$ is uniformly bounded. With all the stipulations above, each $\lambda _i$ is bounded above and below by positive constants. Choosing the minima and maxima of each set produces the *a*- and *b*-values required of part 3 in Theorem 2.
- A straightforward calculation shows that the metric of the constrained Lagrangian $L_c$ corresponding to (16) (with $a=1$) is $g_c=\text {diag}\{I_1,\left[ g(x)\right] ^2\}$. From (b) we know that $g(x)>0$, so this metric is invertible, and thus $L_c$ is regular. Finally, under the stipulations in (a)–(c) $g_p$ is positive definite, which implies that it is invertible.

Under the requirements described above, this class of NHSs satisfies the hypotheses of Theorem 3 and is therefore quantizable with quantum data given in parts 1 and 2 of Theorem 3. A straightforward calculation shows that

24 $$\begin{aligned} R=\frac{I_2I_3}{2I_1}\left( A^{z}_{y}\right) '(x)(f(x))^3=\frac{I_2I_3\left( A^{z}_{y}\right) '(x)}{2I_1\left( I_2+I_3\left( A^{z}_{y}(x)\right) ^2\right) ^{3/2}}. \end{aligned}$$

As an explicit example of this class we consider the following variant of a “nonholonomic free particle”^7^[Sec. 5.6.2] that was numerically simulated in^32^:

25 $$\begin{aligned} L^*=\frac{1}{2}\left( {\dot{x}}^2+2{\dot{y}}^2-{\dot{z}}^2\right) , \quad {\dot{z}}=-\sin (x){\dot{y}}, \end{aligned}$$

with $Q={\mathbb {R}}^{m}\times {\mathbb {S}}^{3-m}$ (here $0 \le m \le 3$). The multiplier (17) in this case is $f(x)=(2-\sin ^2 x)^{-1/2}=(1+\cos ^2 x)^{-1/2}$, and (18) becomes

26 $$\begin{aligned} L^*_{p}=\frac{1}{\sqrt{1+\cos ^2 x}}\left\{ \frac{1}{2}\left( {\dot{x}}^2+2{\dot{y}}^2+{\dot{z}}^2\right) +\sin (x){\dot{y}}{\dot{z}}+{{\mathscr {E}}}^0_c\right\} . \end{aligned}$$

We now verify the stipulations in (a)–(c) above. Relative to (18), (26) has $I_1=1$, $I_2=2$, and $I_3=-1$ (thus (a) is satisfied), and (25) has $A^{z}_y(x)=\sin (x)$. Since $I_2=-2I_3$, here $k=2$, and since $\left( A^{z}_y(x)\right) ^2=\sin ^2 x \le 1$, (20) is satisfied. Furthermore, $A^{z}_y(x)$ and its derivatives are smooth. Thus, (b) is satisfied. Next, since

$$\begin{aligned} 1 \le 1+\cos ^2 x \le 2 \quad \implies \quad \frac{1}{\sqrt{2}} \le f(x) \le 1, \end{aligned}$$

so that (22) holds with $a_1=\sqrt{2}$ and $b_1=1$. All that remains is to check (23). In the present case, this reads $-(-1) \ge (1 \cdot 1)^2/(2+1)^3 = 1/9$, which is true. Thus, all hypotheses of Theorem 3 are verified. The Schrödinger equation $\widehat{H^*}_p(\psi )=E\psi$ is

$$\begin{aligned} -\frac{\hslash ^2}{2}\Delta \psi +\left[ \frac{R\hslash ^2}{12}-(E+f(x){{\mathscr {E}}}^0_c)\right] \psi =0, \quad \text {where} \quad R=-\frac{\cos ^2 x}{\left( 1+\cos ^2 x\right) ^{3/2}}. \end{aligned}$$

This is not explicitly solvable, but various approaches could be used to approximate the solutions. We will not pursue this here. However, we hope that this explicit example illustrates the power of the results arrived at herein—the NHS (25) (and more generally, all members of the family satisfying the stipulations in (a)–(d) above), for which no well-defined quantization was defined prior to this article, has now been quantized. The remaining details (i.e., the functional form of the wavefunctions, the energy spectrum), while cumbersome in some cases, are merely an application of known methods.

## Discussion

Despite the non-Hamiltonian nature of NHSs, we have shown herein that we can successfully quantize abelian Chaplygin, Chaplygin Hamiltonizable NHSs provided they satisfy the hypotheses of Theorem 3 and that we choose the initial wavefunction and restrict the energy state as described at the end of “Background”. Thus, our results represent a significant generalization of the known quantization results for nonholonomic systems, and contain the quantization results of simpler cases as subcases. (When $f(r)=1$ for example—which corresponds to conditionally variational NHSs^18^—the operator (14) reduces to the Hamiltonian operator in^17^.)

The quantum mechanics that results for our work herein, as (14) implies, is driven by a rich interplay of geometry (via the Ricci scalar curvature *R*), mechanics (via the Hamiltonian $H_p$), and phase space volume preservation (recall from the Introduction that Chaplygin Hamiltonizable NHSs preserve phase space volume^7^[Thm. 8.9.1]). In particular, this rich dance plays out on the potential energy stage: the bracketed term in (14) is the effective potential for the associated Schrödinger equation that determines the quantum wavefunctions. Depending on the NHS system, that effective potential will determine the quantum mechanics through the particular mix of geometry, mechanics, and phase space volume preservation implied by the system’s $L_p$ Lagrangian and multiplier *f*. This may lead to interesting properties of the quantum system. For example, in^19^ the associated Hamiltonian system to the particular NHS studied therein featured a constant *R* and $f(r)=1$, and this particular mix resulted in a shift in the ground state energy of the quantized system.

In cases when the NHS under study models a physical system, the rich interplay described above may have physically-relevant consequences. For example, returning to^19^, the aforementioned ground shift involved system parameters that represented the moments of inertia, mass, and diameter of a “molecular wheelbarrow” and its (molecular) wheels. Thus, in that context the geometry and mechanics led to an energy shift that depended on the physical attributes of the system. For other systems the quantum effects of the effective potential in (14) may be more complicated. In^14^, we briefly reviewed the plethora of abelian Chaplygin, Chaplygin Hamiltonizable NHSs studied in the literature, many of which, as we mentioned in the Introduction, model the rolling of rigid bodies on surfaces, which are the macroscopic models of various nanovehicles recently synthesized in laboratories (see^19^ for a brief discussion). The work herein now makes the investigation of the quantum mechanics of all these systems possible, and also allows one to connect that quantum data to the system’s particular physical attributes and the geometry, mechanics, and phase space volume preservation properties of the system’s mathematical model.
